# Multi-Object Positioning and Imaging Based on Single-Pixel Imaging Using Binary Patterns

**DOI:** 10.3390/s22093211

**Published:** 2022-04-22

**Authors:** Wenwen Meng, Dongfeng Shi, Wei Yang, Linbin Zha, Yuefeng Zhao, Yingjian Wang

**Affiliations:** 1 School of Artificial Intelligence and Big Data, Hefei University, Hefei 230601, China; mengww@hfuu.edu.cn; 2 Advanced Laser Technology Laboratory of Anhui Province, Hefei 230037, China; lx1993@mail.ustc.edu.cn (W.Y.); lb456@mail.ustc.edu.cn (L.Z.); wyj@aiofm.ac.cn (Y.W.); 3 Key Laboratory of Atmospheric Optics, Anhui Institute of Optics and Fine Mechanics, Hefei Institutes of Physical Science, Chinese Academy of Sciences, Hefei 230031, China; 4 University of Science and Technology of China, Hefei 230026, China; 5 Collaborative Innovation Center of Light Manipulations and Applications, Shandong Normal University, Jinan 250358, China; yuefengzhao@sdnu.edu.cn

**Keywords:** single-pixel imaging, binary modulation, multi-object positioning

## Abstract

Single-pixel imaging (SPI) is a new type of imaging technology that uses a non-scanning single-pixel detector to image objects and has important application prospects and value in many fields. Most of the modulators currently used in SPI systems are digital micromirror device (DMD) modulators, which use a higher frequency for binary modulation than other alternatives. When modulating grayscale information, the modulation frequency is significantly reduced. This paper conducts research on multiple discrete objects in a scene and proposes using binary patterns to locate and image these objects. Compared with the existing methods of using gray patterns to locate and image multiple objects, the method proposed in this paper is more suitable for DMD-type SPI systems and has wider applicability and greater prospects. The principle of the proposed method is introduced, and the effectiveness of the method is experimentally verified. The experimental results show that, compared to traditional SPI methods, the number of patterns required by the proposed method is reduced by more than 85%.

## 1. Introduction

Single-pixel imaging (SPI) [1,2,3,4,5,6,7,8,9,10,11,12,13,14,15,16,17,18,19,20,21,22,23,24,25,26,27] is a new method of capturing images of objects using a non-scanning single-pixel detector. The core idea of SPI is briefly described as follows: First, the known illumination patterns are projected onto the object, then the total intensity of the reflected light is measured by a single-pixel detector, and finally, the correlation between the projected patterns and the recorded signals is used to reconstruct the image. SPI is a technology that obtains a high pixel resolution at the expense of the temporal resolution. Due to its characteristics of high signal-to-noise ratio (SNR) imaging under low signal light [2] and wider spectral imaging, SPI has been used in multi-spectral imaging [3,4,5], three-dimensional imaging [6,7], gas imaging [8], terahertz imaging [9,10], and so on.

In order to reconstruct an image with a high pixel count, SPI needs to project a large number of patterns, and the number of patterns increases as the number of image pixels increases. Improving the real-time performance of SPI is one of the most important research topics in this field. Recently, the application of deep learning with convolutional autoencoder networks can recover real-time 128 × 128 pixels’ video at 30 fps [11]. Ref. [12] used compressed sensing technology to achieve a resolution of 256 × 256 pixels with a frequency of 11 frames. An SPI scheme using a light-emitting diode (LED)-based high-speed illumination module was proposed, and an outstanding frame rate of 1000 fps with 32 × 32 pixels’ resolution was achieved [13]. This method, which is based on forward modulation, can work only under active illumination conditions. At the same time, the imaging resolution is limited by the LED array, and it is very difficult to achieve a resolution of over megapixels as with spatial light modulators (SLMs), such as a DMD with a 2560 × 1600 pixels’ resolution. Due to the inverse relationship between the imaging frame rate and the imaging pixel resolution, increasing the imaging frame rate requires a reduction in the imaging pixel resolution. For example, under the same compression sampling rate, the imaging frame rate is 1000 fps with a 32 × 32 pixels’ resolution, whereas the imaging frame rate is 15.6 fps with a 256 × 256 pixels’ resolution. It can be seen that for SPI systems with a higher pixel count, the number of patterns required becomes very large, and the time consumption becomes unacceptable, which severely limits the application of SPI in multiple fields, such as imaging fast-moving objects.

In an SPI system, the light modulator is a key device. Matthew P. Edgar [1] summarized the principles and prospects of SPI and studied the types of light modulators that can be used for SPI. It was pointed out that a DMD with high-speed modulation capability in SPI is the most common and ideal choice due to its superior modulation rates in excess of 22 kHz [1]. However, this setup still cannot meet the need of fast real-time SPI. When the modulation frequency of the optical modulator is limited, reducing the number of projected patterns becomes an effective way to improve the efficiency of SPI. In recent years, research on the use of orthogonal bases as patterns has led to the continuous development of SPI technology. Studies have shown that the number of patterns using an orthogonal basis is far fewer than the number of patterns using a non-orthogonal basis for SPI. Orthogonal-based patterns are currently used in two of the most popular techniques: Hadamard and Fourier SPI [19,20,21,22]. Aiming at the relatively small area of the imaging object in the scene, an adaptive regional Fourier SPI method is proposed in the literature [23]. The core is to first use the Fourier slice theorem to locate the object in the vertical and horizontal directions, then project the Fourier patterns only on the object area, and finally obtain the Fourier spectrum of the object area and reconstruct the image. Compared with the traditional SPI method that needs to image the entire scene, this method significantly reduces the number of projected patterns and improves imaging efficiency. The method is further improved in [24], which realizes the positioning of multiple objects and high-efficiency Fourier SPI, and reduces the time consumed by approximately 90%; that is, the required projection patterns are reduced by 90%. Both the above methods [23,24] use the Fourier slice theorem to complete object localization and use Fourier SPI technology to complete the imaging. Specifically, these methods employ the Fourier slice theorem to obtain the projection curve and determine the position of objects, and then use Fourier SPI to sample only the Fourier spectrum of the object area, so a large number of invalid background area samples will be discarded. Compared with the traditional SPI technology, which samples only low-frequency component information [22], the proposed method can obtain complete object spectrum information without losing object information at a low spatial sampling rate (SR).

It is worth noting that the frequency of DMD-modulated binary patterns can reach more than 22 kHz, and the frequency of 8-bit grayscale patterns is approximately 250 Hz [1,21]. Many existing studies on SPI use DMD as the optical modulator. In other words, most SPI systems are DMD-type systems [1]. However, Fourier SPI requires gray patterns. To overcome the low modulation rate of DMD for gray patterns, Ref. [25] proposed a spatial dithering technique to make full use of the frequency of DMD binary modulation, but at the expense of spatial resolution. We [21] used the method of detected signal weights to improve DMD-type Fourier SPI efficiency without the loss of spatial resolution, but still failed to make full use of the DMD binary modulation frequency. At present, there is no Fourier SPI technology that can fully utilize the performance of DMD in terms of the binary modulation frequency and spatial resolution. Therefore, unlike the gray patterns used in [23,24], the binary patterns used for object positioning and imaging is a very meaningful research work.

As another popular technology, Hadamard SPI technology can fully utilize the modulation frequency and spatial resolution of DMD due to the use of binary patterns [1,19]. It has the most application value and prospect in SPI systems based on DMD modulators and is one of the most widely used technologies for SPI of DMD modulators. Ref. [19] compared SPI systems based on Hadamard and Fourier patterns and concluded that Hadamard patterns are more efficient and more suitable for SPI systems with DMD modulators. Therefore, it is necessary to study object positioning and imaging technology using binary patterns to optimally match the advantages of DMD-type SPI systems. We proposed using binary patterns to achieve the rapid positioning and tracking of a single moving object, but no imaging research has been carried out [15,16,17]. Furthermore, we also proposed Radon SPI [18], which uses binary patterns to obtain the Radon spectrum of the object and then inversely transforms the spectrum to obtain the object image. The curve of each angular position in the Radon spectrum corresponds to the projection curve at each angle and can be used to position multiple objects. Aiming at the relatively small area of the objects in the scene, different from the existing literature [23,25], combined with our proposed Radon SPI technology, this paper proposes a new multi-object positioning and imaging based on binary patterns that can make full use of the performance of the DMD modulator and can achieve multi-object positioning and imaging with a few binary patterns. The proposed method uses binary patterns, so it is more suitable for applying the most common DMD-type SPI system. Section 2 introduces the method of this paper, Section 3 introduces the experimental results, and Section 4 is the conclusion.

## 2. Theory and Principle

Different from the traditional SPI method, the proposed method used in this paper needs to project patterns only in the object area and does not need to project patterns in the entire scene. Therefore, fewer sampling points in the effective object area are needed, which can effectively improve the imaging efficiency. The significant difference from the existing similar methods [23,24] is that the method proposed in this paper is based on binary patterns; thus, the performance of the DMD modulator is fully utilized.

**Figure 1:**
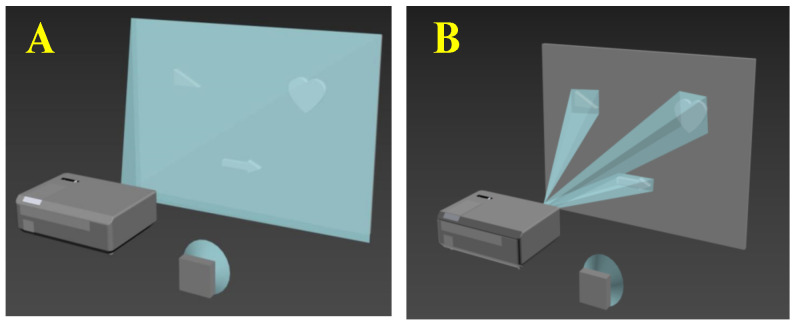
Comparison of two single-pixel imaging methods: (**A**) traditional single-pixel imaging; (**B**) positioned single-pixel imaging.

For the sparse scene where the object area occupies a small part of the scene area, Figure 1 shows two types of SPI diagrams. Figure 1A is a conventional SPI technology, and Figure 1B is an area-positioning SPI technology. SPI technology based on area-positioning samples only the area containing the object and discards the sampling of a large number of background areas. Therefore, compared with the traditional method of sampling the entire scene area, this method is more efficient and achieves very good performance in sparse scenes. The method in this paper mainly includes two steps. The first step is to locate multiple objects combined with our proposed Radon SPI [18], and the second step is to sample and restore the object part combined with the Hadamard SPI.

### 2.1. Multi-Object Positioning Method

The one-dimensional vector *T_k_* from the two-dimensional Hadamard transform is projected along the $\theta_{m}=\left(0,\;\pi \right]\;$direction to obtain a two-dimensional illumination pattern, which can be expressed using the following formula:

(1) $$S_{\theta_{m,k}}(x,y)=\sum_{L}T_{k}(L)\delta (x\cos\theta_{m}+y\sin\theta_{m}-L)$$

where *k* is an index value and (*x*, *y*) represents the object space coordinate. According to the characteristics of the *δ* function, it can be known that when (*x*, *y*) satisfies the formula $x\cos\theta_{m}+y\sin\theta_{m}=L,\;\delta =1$; otherwise, *δ* = 0. Traversing the entire coordinate system can yield two-dimensional illumination patterns. According to the above analysis, it can be found that the binary patterns are obviously different from the gray patterns used in [23,24]. The Radon transform of scene image *f* (*x*, *y*) can be expressed by the following formula [18]:

(2) $$F(L,\theta )=\sum_{x,y}f(x,y)\delta (x\cos\theta +y\sin\theta -L)$$

A one-dimensional curve $F\;\left(L,\;\theta \right)$ for the determined angle *θ* and all *L* represents the projection curve of the scene at angle *θ*. The constructed illumination patterns are used to illuminate the scene to detect the total echo signal from the scene. The detection signal can be expressed as follows:

(3) $$I_{k}=\sum_{x,y}S_{\theta_{m},k}(x,y)f(x,y)$$

Formula (4) is obtained by substituting Formula (1) into Formula (3) and using the properties of Formula (2); the results are as follows:

(4) $$I_{k}=\sum_{x,y}\sum_{L}T_{k}(L)f(x,y)\delta (x\cos\theta_{m}+y\sin\theta_{m}-L)=\sum_{L}F(L,\theta_{m})T_{k}(L)$$

According to the above formula, the detection signal is equivalent to the effect of the projection curve of the scene at angle θm and a one-dimensional vector of the Hadamard matrix. Therefore, a series of binary patterns at different projection angles are used to illuminate the scene, and the echo signals are detected. The SPI algorithm can be used to obtain the projection curve of the scene at projection angle *θ_m_*. The calculation formula can be expressed as follows:

(5) $$F(L,\theta_{m})=\sum_{L}I_{k}T_{k}(L)$$

Scene projection curves at different angles can be obtained by changing the projection angle of the binary patterns. After obtaining the projection curve, it is subjected to two-dimensional back-projection to obtain the two-dimensional projection image $O_{\theta_{m}}\left(x,\;y\right)$ of the scene at angle $\theta_{m}$ which can be expressed by the following formula:

(6) $$O_{\theta_{m}}(x,y)=\sum_{L}F(L,\theta_{m})\delta (x\cos\theta_{m}+y\sin\theta_{m}-L)$$

The multiple-angle two-dimensional projection images *O_θ_m__*, *m* = 1,…, *i* are obtained from the above formula, and *i* represents the number of projection angles. The interval between each angle can be expressed as follows:

(7) Δθ=π/i

Thresholding is performed on the obtained two-dimensional projection images to obtain the object distribution area. The threshold value is selected as follows:

(8) $$\varepsilon =0.01\max\left\{O_{\theta_{m}}\right\}$$

The selection of threshold is very important. If the selection value is large, there is a high probability that the object selection will be incomplete and some object areas will be lost. However, if the selected threshold is too small, it may be disturbed by factors such as noise and complex background. The selection of 0.01 value is only a choice made by experience. Thresholding two-dimensional projection images according to the above threshold value yield the following:

(9) $$B_{\theta_{m}}(x,y)=\left\{\begin{array}{c} 1,\;S_{\theta_{m}}(x,y)>\varepsilon \\ 0,\;S_{\theta_{m}}(x,y)\leq \varepsilon \end{array}\right.$$

By performing the AND operation on the binary images obtained by the above operation, an object area image is obtained as follows:

(10) $$A_{c}=\underset{m}{\cup }B_{\theta_{m}}$$

The object area can be obtained by the above formula. The spatial SR of the number of isolated areas is equal to the number of objects *j* in the scene. By calculating each object region, each regular rectangular object region can be obtained. The starting coordinates, length, and width parameters of each object area can be calculated using the following formula:

(11) $$\left\{\begin{array}{l} X_{j0}=\min(X_{Aj})\\ Y_{j0}=\min(Y_{Aj})\\ W_{Aj}=\max(X_{Aj})-\min(X_{Aj})\\ H_{Aj}=\max(Y_{Aj})-\min(Y_{Aj}) \end{array}\right.$$

In the above formula, $X_{j0}$ and $Y_{j0}$ represent the starting coordinate position of area $SR_{j}$*,* $X_{Aj}$ and $Y_{Aj}$ represent the area coordinates of object *j*, min and max represent the calculated minimum and maximum values, and $W_{Aj}$ and $H_{Aj}$ represent the width and height of the area $SR_{j}$.

The number of patterns used at each projection angle is P, and the total number of patterns required during object positioning is Q=i×P. If the scene is *N* × *N* pixels, the spatial SR for positioning is $\gamma_{1}=Q/N^{2}$.

A single object needs only horizontal and vertical projection angles to determine its position. If there are multiple objects, multiple projection curves at different angles are required to accurately obtain the area position of each object. The patterns used to locate multiple objects have binary properties, so the proposed method can make full use of the properties of the DMD modulator.

**Figure 2:**
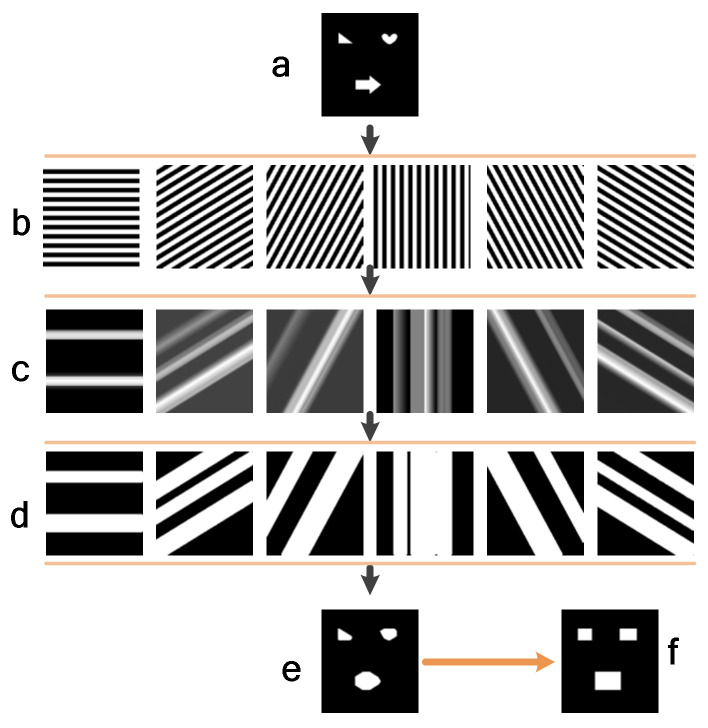
Multi-object positioning flowchart: (**a**) is a scene including three geometrically shaped target objects, namely, a triangle, a heart, and an arrow; (**b**) is the binary patterns at different projection angles; (**c**) is the two-dimensional projected grayscale images of the scene at different projection angles; (**d**) is the binary images obtained by thresholding the grayscale images (**c**); (**e**) is the position of the target object region obtained by performing the AND operation on the binary images (**d**) and then processing to obtain the regular target object region (**f**).

Figure 2 shows the process of multi-object positioning. A two-dimensional *N* × *N* Hadamard matrix is generated, and then the data of each column are projected at different angles to obtain two-dimensional projection patterns of *N* × *N*. Finally, the scene is illuminated with the projection patterns. The second line shows the binary patterns at different projection angles. The third line is the two-dimensional projection images of the scene at different projection angles calculated by Formula (6). The fourth line is the binary projected images obtained by performing thresholding on the images obtained in the third line. In the last row, (e) is the object area obtained by performing the *AND* operation, and (f) is obtained by coordinate processing.

### 2.2. Image Reconstruction

**Figure 3:**
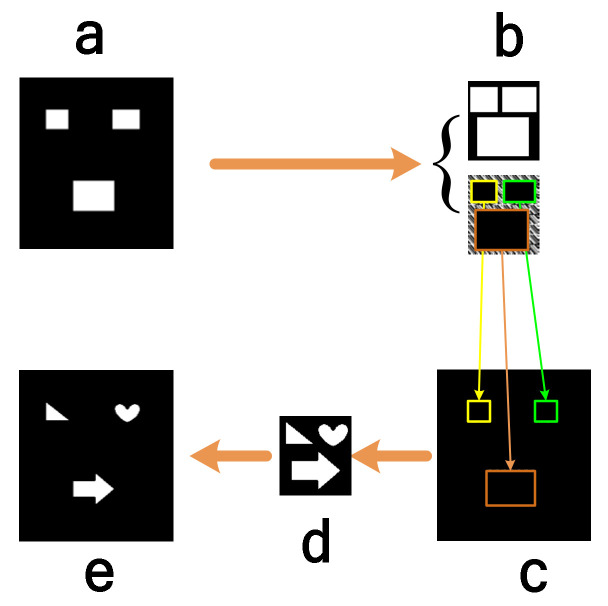
Object reconstruction process: (**a**) is the entire scene area positioned to multiple parts; (**b**) arranges each closed rectangle in (**a**) together to form a rectangle that covers the integration area and generates Hadamard patterns based on the object integration area rather than on the entire scene area; (**c**) repositions the three matrices corresponding to the illuminated Hadamard patterns in (**b**) back to the area of the entire scene; (**d**) projects the Hadamard illumination patterns onto the scene, uses a single-pixel detector to measure the light intensity, and obtains the object image by a reconstruction method; and (**e**) is a complete scene image obtained by dividing the integrated object image into several parts and positioning these parts back to the scene.

When the regular area distributions of multiple objects are obtained, the arrangement is adjusted according to the multiple areas of the objects, and the obtained arrangement area is consistent with the Hadamard illumination matrix area. The operation flowchart is shown in Figure 3. In the process of imaging, Hadamard SPI technology is used, and a detailed description of the technology can be obtained by referring to [1,19]. It can be seen that this paper uses binary patterns in multi-object positioning and imaging, so it can make full use of the performance of DMD modulation, which is well suited for the current common DMD-type SPI system.

The steps of image reconstruction are as follows:

Step 1. The scene HR of the entire area is shown in Figure 3a. The width, height and coordinate point position of the upper-left corner of each rectangle in the coordinate system are recorded, and then those rectangles are put close to each other to generate a bounding rectangle (BR), as shown in Figure 3b. The method of generating a BR is very flexible, and the ultimate goal is to ensure that the BR is as small as possible. The method of this article is consistent with that used in [24]. However, because the Hadamard SPI method is utilized in this paper, the shape of the BR must be square, and the length *M*, *M*/12 or *M*/20 is a power of 2.

Step 2. As shown in Figure 3b, Hadamard patterns are generated based on the stitching area BR rather than the entire scene area HR. The patterns are separated into several parts, and then these parts are located back to the area HR according to the coordinates as shown in Figure 3c.

Step 3. Figure 3c shows that one Hadamard pattern is irradiated on each object in the scene, and a single-pixel detector is used to measure reflected light from multiple objects.

Step 4. Using detection intensities and patterns, the reconstructed image $O_{o}\left(x,\;y\right)$ is obtained (Figure 3d).

Step 5. The reconstructed image is divided into several parts, and these parts are located back into the HR, as shown in Figure 3e. The reconstructed image $O_{o}\left(x,y\right)$ is divided into several parts, which are represented by $O_{o,j}\left(x,y\right)$ where *j* represents the number of objects. In Figure 3e, *j* equals 3. The reconstructed scene image $O_{r}\left(x,y\right)$ can be expressed as:

(12) $$O_{r,j}(X_{j0}+x,X_{j0}+y)=O_{o,j}(x,y).\left\{\begin{array}{c} 0\leq x\leq W_{Aj}\\ 0\leq y\leq H_{Aj} \end{array}\right.$$

Assuming that the resolution of the scene HR is *N* × *N* and the number of projection patterns used for imaging is *G = M* × *M*, then the spatial *SR* at imaging is *γ_2_ = G/N*^2^. Compared with [24], which employs gray patterns, the method can fully use the modulator frequency of DMD and has wider value and prospects. The total spatial *SR* is determined by the number of patterns used in positioning and imaging and the number of HR pixels in the scene. Since the number of patterns required for object positioning is small, when the BR occupies a relatively small number of pixels compared to the area scene HR, the number of samples required to acquire object information is small. The total spatial *SR* is equal to *γ = γ*_1_ *+ γ*_2_ *=* (*Q + G*)*/N*^2^.

## 3. Results

### 3.1. Numerical Experiment

**Figure 4:**
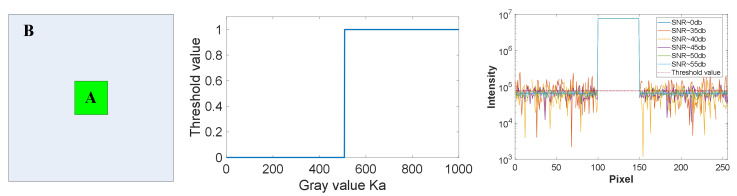
Numerical simulation experiments under different contrasts and SNRs. (**Left**) part A is the area of uniform grayscale object, and part B is the area of uniform grayscale background. (**Middle**) threshold conditions of object area under different gray values. (**Right**) projection curve obtained under different SNRs.

Simulation experiments are conducted on objects with different contrasts, as shown in Figure 4. The figure on the left is a schematic of a scene with the presence of a background. Part B is assumed to be a background region with consistent gray values, the size of the region is Sb×Sb pixels and the gray value of the background region is Kb. Assume that part A is the object region with the consistent gray values, the size of the region is Sa×Sa pixel, and the gray value of the background region is Ka. The one-dimensional cumulative value of the area containing the object is $Ta=Kb\times \left(Sb-Sa\right)+Ka\times Sa$, and the cumulative value of the background is Tb=Kb×Sb. According to the criterion, the threshold processing can be carried out to obtain the area where the object is located only when the equation 0.01×Ta>Tb is satisfied. The middle figure shows the numerical simulation results of different contrast objects. Set Sb=256, Kb=1, Sa=50, Ka is the change value, take x direction cumulative curve for example, and other projection cumulative curve has similarity. It can be seen that the object region can only be recognized when the gray value of the object is 508. Therefore, the next focus of our work is to improve the applicability of the algorithm in this paper under the condition of low contrast.

When multiple objects with different gray values exist, the above conditions still need to be met for each object to be detected. If an object cannot meet the conditions of detection in the projection curve, its positioning imaging cannot be realized. On the contrary, if the object meets the conditions, its positioning imaging can be realized. When the scene has a background, it will affect the location imaging results. Different backgrounds have different effects, so perhaps we can use methods similar to those in [15]. Firstly, the background value is measured, and then the measured value containing the object is subtracted to obtain the equivalent detection value containing only the object.

We make a simulation study on the noise of measurement signal. According to the above parameters, Ka value was set to 600, and Gaussian white noise with a different SNR was added to the detection value. The obtained projection curve results are shown on the right in Figure 4. The obtained one-dimensional projection curves were calculated using root mean square error (RMSE), and the RMSE obtained under SNR of 35 dB, 40 dB, 45 dB, 50 dB, and 55 dB were 0.013, 0.006, 0.003, 0.002, and 0.001, respectively. The area of the object is determined by the threshold. The true value is 50 pixels, and the number of pixels occupied by the object area under SNR of 35 dB, 40 dB, 45 dB, 50 dB, and 55 dB is 135, 114, 104, 72, and 50, respectively. It can be seen that when the SNR is better than 55 dB, accurate object area parameters can be obtained.

### 3.2. Multi-Object Positioning Experiment

The proposed technique is studied by an experimental system, which is described as follows. A 10 W white LED serves as the light source. A DMD system (Texas Instruments Discovery V7100 with 1024 × 768 micromirrors) is used to generate illumination patterns. A single-pixel detector (SD, Thorlabs PMT-PMM02) and data acquisition system (Pico 6407 with a temporal *SR* of 200 MS/s) are employed for light detection and data acquisition, respectively. The light enters a lens and then passes through the DMD, which provides the patterns, which are projected to the object by a lens with a focal length of 125 mm. The reflected light from the object is collected by a lens with a focal length of 100 mm and then detected by the SD. Next, the intensity values are sent to a computer through a data acquisition system (DAS). The 3 × 3 mirrors of the DMD are combined into a pattern cell that corresponds to an image pixel, and the intermediate 768 × 768 mirrors are utilized in the experiment. Thus, the resolution of the image is 256 × 256 pixels.

**Figure 5:**
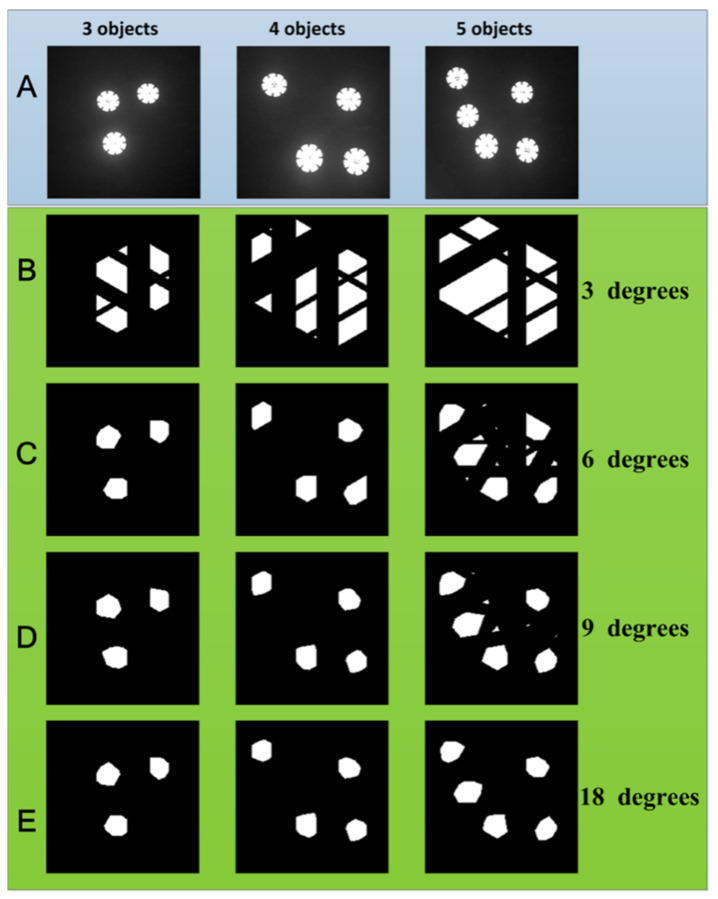
The positioning results of multiple objects with different numbers of projection angles. The first, second and third columns are the cases of three, four, and five objects, respectively; (**A**) is the original object image, and (**B**–**E**) represent the positioning results of the object areas at three, six, nine, and eighteen projection angles, respectively, and the corresponding separation angles are 60, 30, 20, and 10 degrees, respectively. The white areas are the located object areas.

In the first multi-object positioning experiment, three, four, and five circular objects (buttons) were used. The images are shown in Figure 5A. The experiments on the positioning effect of multiple objects are performed using different numbers of projection angles; the results are shown in Figure 5B–E. In this experiment, 256 projection patterns were used to obtain the projection curves of the object at each angle. When the number of objects is small, a relatively small number of projection angles can be used to obtain a more accurate positioning object area; when the number of objects is large, more projection angles need to be used to locate more accurate object areas. The number of objects has a non-linear relationship with the number of required projection angles. For example, in this experiment, when three and four objects exist, six angles of the projection curve can be used to locate the accurate object area; when the number of objects is five, eighteen projection angles must be used to locate the object area. The required projection angle is not only related to the number of objects, but also related to the relative position between objects. If five objects are arranged in a row along the *x*-axis, only two projection angles of 0 degrees and 90 degrees are needed to realize positioning. If the arrangement is like that in this paper, eighteen projection curves are required. Because the relative position between objects is very complex, it needs to be analyzed in specific scenes.

**Figure 6:**
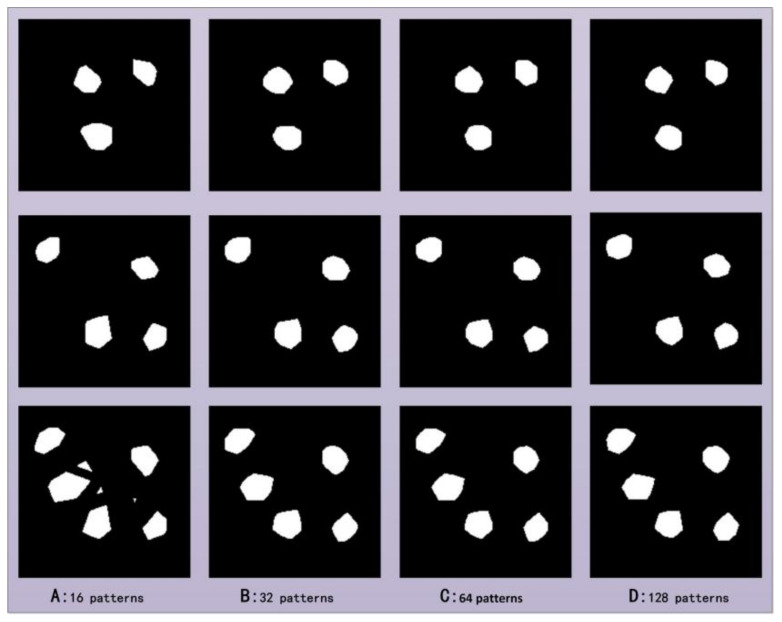
Multi-object positioning results at different sampling rates. The first, second and third rows indicate the positioning results of three, four, and five objects at different sampling rates, respectively; (**A**–**D**) represent the positioning results, and the numbers of projection patterns for each angle are 16, 32, 64, and 128, respectively.

In the second experiment, the number of projection angles was 18, and the results of multi-object localization were studied using different numbers of patterns. The number of patterns required to obtain the projection curve for each angle is set to a variable value, which is equal to 16, 32, 64, and 128. The experimental results are shown in Figure 6. It can be seen that to obtain accurate object area information, when the number of objects is small, a small number of patterns is required. As the number of objects increases, the number of patterns required increases accordingly. In this set of experiments, the number of patterns for each angle required for three or four objects is sixteen, and the number of patterns required for five objects is thirty-two. Similar to the results of the above group, to obtain an accurate object area, the spatial *SR* and the number of objects increase non-linearly.

Through the above analysis, using eighteen projection angles and thirty-two patterns at each angle can achieve accurate positioning of at most five objects. The spatial *SR* at the positioning stage is:

(13) $$\gamma_{1}=18\times 32/256^{2}\approx 0.88\%$$

It can be seen that the spatial *SR* is very small in the process of object positioning. However, this process selects the effective area and discards invalid areas without objects. This operation is very effective when the object area is relatively small compared with the scene area, which can greatly reduce the samples needed for imaging. This process improves the efficiency of SPI.

### 3.3. Multi-Object Imaging Experiment

**Figure 7:**
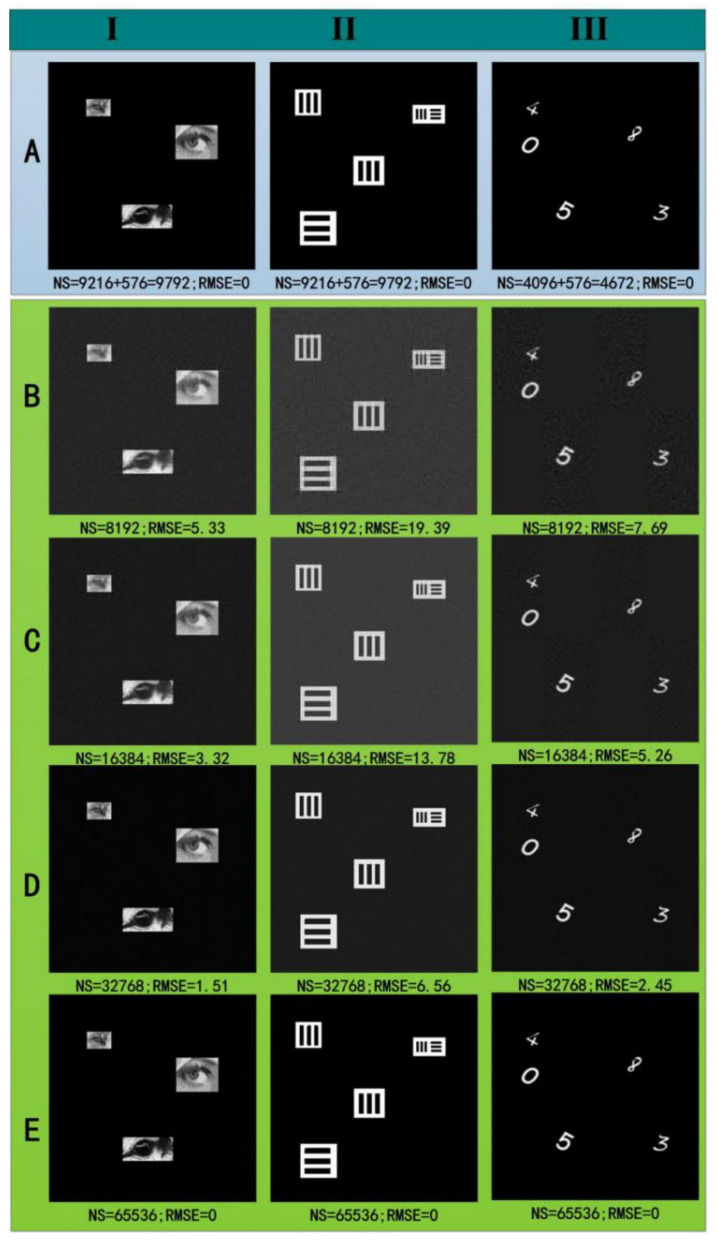
Multi-object imaging comparison results. (**A**) shows the results obtained by using the proposed method in this article under different NS 9792, 9792, and 4672, respectively; (**B**–**E**) are the results obtained by the traditional Hadamard single pixel imaging method under different NS 8192, 16,384, 32,768, and 65,536, respectively.

The following experiment uses the technology proposed in this paper to image multiple separated objects. When the positioning of the objects is completed, the Hadamard SPI scheme is used to image the multiple separated objects. The results are shown in Figure 7. In this experiment, three groups of different types of multiple objects are used: The first group (the I column) is three separated gray objects, and the second and third groups (the II column and III column) are four and five binary separated objects. The objects in the third group occupy the smallest effective area. The entire scene is 256 × 256 pixels. For the first and second groups, the objects can be represented by an equivalent 96 × 96 pixels’ area, and the objects in the third group can be represented by an equivalent 64 × 64 pixels’ area.

In the experiment, the root-mean-square error (*RMSE*) parameter [12] is used to evaluate the quality of the restored images and is expressed as follows:

(14) $$RMSE=\sqrt{\sum_{x,y}{\left[O_{r}(x,y)-O_{s}(x,y)\right]}^{2}/N^{2}}$$

where *O_r_*(*x,y*) and *O_s_*(*x,y*) are the values of the (*x*,*y*)-th pixel in the reconstructed image and reference image under full sampling, respectively. The smaller the *RMSE* is, the better the recovered image quality is.

The imaging results are shown in Figure 7. Line A shows the results obtained by using the proposed method in this article. NS indicates the number of projection patterns used and consists of two parts: The first part is the number of patterns required to image, and the second part is the number of patterns needed to position the multiple objects. It can be seen from the results that NS is relatively low, and the spatial *SR* of the method used in this paper decreases as the number of objects decreases. Figure 7B–E shows the results obtained by the traditional Hadamard single-pixel imaging method [19]. Compared with traditional SPI, the proposed method in this paper can obtain high-quality images at a small spatial *SR*. The spatial *SRs* of the first and second groups are approximately 15%, and the spatial *SR* of the third group is approximately 7%. Compared with the traditional method, the number of samples required by this method in the first and second groups is reduced by approximately 85%. When the number of multiple objects is relatively small, as in the third group, the number of samples is reduced by approximately 93%. Because there are few projection patterns used in the multi-object localization stage, the imaging of separate objects requires most of the samples. The number of samples required for imaging is directly related to the proportion of separated objects in the overall scene. When the proportion of separated objects is relatively small, the number of samples required by the proposed method will be smaller than the number of samples required by the traditional methods.

The method in this paper uses binary patterns in the two processes of multi-object positioning and imaging, so it is especially suitable for the current common DMD-type SPI systems.

## 4. Conclusions

To effectively reduce the number of projection patterns required by an SPI system, this paper proposes using binary patterns to position and image multiple discrete objects in a scene. Compared with the existing direct SPI method [3,4,5,6,7,8,9,10], the proposed method can effectively reduce the number of samples. In the experiment, this method can reduce the number of samples by more than 85% compared with the traditional method. Of course, the specific reduction has a close relationship with the proportion of discrete objects in the entire scene. The smaller the proportion is, the smaller the spatial *SR*, and vice versa. Compared with existing similar methods using gray patterns [21,22], the proposed method in this paper uses binary patterns. The common SPI systems currently use a DMD as a modulator, and binary patterns can fully utilize the performance of the DMD, which is more in line with the current DMD-type SPI systems. The method proposed in this paper has important application prospects and value when there are multiple discrete objects in a scene. Combining these algorithms [28,29,30] can further improve the efficiency of the method.

## Data Availability

Not applicable.

## Abbreviations

The following abbreviations are used in this manuscript:

SPI	single-pixel imaging
SR	spatial *SR*
BR	bounding rectangle
RMSE	root-mean-square error
DMD	digital modulating devices
